# Differential regulation of brain‐derived neurotrophic factor (BDNF) expression in sensory neuron axons by miRNA‐206

**DOI:** 10.1002/2211-5463.12581

**Published:** 2019-01-16

**Authors:** Shiva Shrestha, Monichan Phay, Hak Hee Kim, Pedram Pouladvand, Seung Joon Lee, Soonmoon Yoo

**Affiliations:** ^1^ Nemours Biomedical Research Alfred I. duPont Hospital for Children Wilmington DE USA; ^2^ Department of Biological Sciences University of Delaware Newark DE USA; ^3^ Gene Therapy Program Perelman School of Medicine University of Pennsylvania Philadelphia PA USA; ^4^ Department of Biology Pennsylvania State University‐Brandywine Media PA USA; ^5^ Department of Biological Sciences University of South Carolina Columbia SC USA

**Keywords:** 3′ UTR variants, BDNF mRNA, brain‐derived neurotrophic factor, differential regulation, dorsal root ganglion, miRNA‐206, mRNA localization, sensory neuron, small non‐coding microRNA

## Abstract

Distinct subcellular localization and subsequent translational control of 3′ UTR variants of mRNA encoding brain‐derived neurotrophic factor (BDNF) are critical for the development and plasticity of neurons. Although the processes that lead to preferential localization of BDNF have been well studied, it is still not clear how neurons ensure differential BDNF production in a spatial‐specific manner. Here, we identified that microRNA (miRNA)‐206 has the potential to specifically regulate BDNF with a long 3′ UTR without affecting its short 3′ UTR counterpart. Overexpression of miRNA‐206 in sensory neurons resulted in a 30% and 45% reduction of BDNF protein expression in the cell bodies and axons, respectively. The work described in the present study indicates that miRNAs can differentially and specifically regulate the expression of transcript variants with different localization patterns.

**Abbreviations**
BDNF: brain‐derived neurotrophic factor
ddPCR: droplet digital PCR
DRG: dorsal root ganglion
ErbB3: receptor tyrosine‐protein kinase ErbB family‐3
FRAP: fluorescence recovery after photobleaching
GFAP: glial fibrillary acid protein
H1F0: H1 histone family member 0
MAP2: microtubule‐associated protein 2
miRNA: microRNA
ROI: region of interest

Brain‐derived neurotrophic factor (BDNF) is a secreted protein of the neurotrophin family that plays crucial roles in the development and survival of neurons, growth of neuronal processes and synaptic plasticity 1, 2, 3, 4. An aberrant level of BDNF expression is closely associated with the pathophysiology of numerous neurological disorders including Alzheimer's disease, Parkinson's disease and Huntington's disease 5, 6, 7, 8, 9. Therefore, the expression of BDNF is tightly regulated by many processes at both the transcriptional and post‐transcriptional levels. Although much is known about how the levels of *BDNF* transcript are regulated by diverse promoters, DNA methylation and alternative splicing, detailed mechanisms of post‐transcriptional regulation, such as subcellular localization and translation, still remain to be clarified.

At the post‐transcriptional level, the primary BDNF transcript generates two different 3′ UTR variants of *BDNF* mRNA, a short (0.35 kb) or a long 3′ UTR (2.9 kb), as a result of two alternative polyadenylation signals present in the 3′ coding exon 1, 10, 11. The distinct 3′ UTR variants of *BDNF* mRNAs provide a means to control BDNF expression via mRNA localization and/or translational control 10, 12. The short 3′ UTR variant of *BDNF* mRNA is reported to be restricted in the soma and the long variant is preferentially transported to dendrites, contributing to the activity‐dependent rapid increase in local expression 12. These studies indicate that the 3′ UTR of *BDNF* mRNA may play important roles in both mRNA localization and regulation of translation.

Recently, small non‐coding RNAs such as microRNAs (miRNAs) have been found to mediate the control of key genes involved in the nervous system, suggesting that miRNAs have a role as key regulators in BDNF expression at the post‐transcriptional level. However, it is still unclear how translation of these 3′ UTR variants of the transcripts is differentially regulated in a spatial‐specific manner. The targeting sites that miRNAs recognize and make base‐pairing are often present within the 3′ UTR of target mRNAs 13, 14, 15. Recently, Lee *et al*. 16 showed that aberrantly high levels of miRNA‐206 in Alzheimer diseased brains are directly involved in the pathogenesis of Alzheimer disease by targeting the 3′ UTR of *BDNF* mRNA, suggesting that BDNF expression is post‐transcriptionally regulated by miRNA‐206. Therefore, we hypothesized that the long 3′ UTR variant of *BDNF* mRNA could be subjected to the differential regulation by specific miRNAs from the counterpart with a short 3′ UTR in a spatial‐specific manner.

In the present study, we show that the long 3′ UTR variant of *BDNF* mRNA is endogenously present in axons of sensory neurons, although with very low abundance. The miRNA‐206 specifically regulated this variant of *BDNF* mRNA in axons. The transfection of miRNA‐206 in primary culture of dorsal root ganglion (DRG) neurons from adult rats resulted in a significant reduction of BDNF protein. Overall, our findings demonstrate a unique ability of miRNA‐206 in the selective regulation of intra‐axonal translation via the targeting specific sequences only present in localizing *BDNF* mRNA with a long 3′ UTR.

## Materials and methods

### Animal use

Animal procedures were approved by the Institutional Animal Care and Use Committees (IACUC) at the Nemours/Alfred I. duPont Hospital for Children, and the experiments were conducted under the IACUC at Alfred I. duPont Hospital for Children. Sprague–Dawley rats (weighing 150–225 g) were killed by asphyxiation with CO_2_ using compressed sources of gas. When dead, rat tissues, including the brain, DRG and sciatic nerves, were surgically removed from the rat and the carcasses were disposed in conformance with regulations.

### RNA extraction and quantification

Total RNA from rat L4–L6 DRGs and brain were isolated using phenol–chloroform extraction followed by ethanol precipitation. To isolate the axoplasm from the rat sciatic nerve, a mechanical squeezing method was used, as described previously 17. Briefly, the sciatic nerves were cleaned from the surrounding connective tissues using ultrafine forceps in cold phosphate‐buffered saline. The sciatic nerve was cut into segments of approximately 10 mm in length using a surgical blade. Then, the axoplasm was carefully squeezed manually using a pestle fit into a 1.5 mL microcentrifuge tube containing the lysis buffer on ice. Nucleic acids were isolated with the RNAqueous™‐Micro Total RNA Isolation kit (Ambion, Austin, TX, USA). The concentration of RNA extracted was determined by the VersaFluor™ fluorometer (Bio‐Rad, Hercules, CA, USA) using RiboGreen™ reagent (Invitrogen, Carlsbad, CA, USA) and then stored at −80 °C until use.

### cDNA synthesis and axoplasm RNA purity

**Figure 1:**
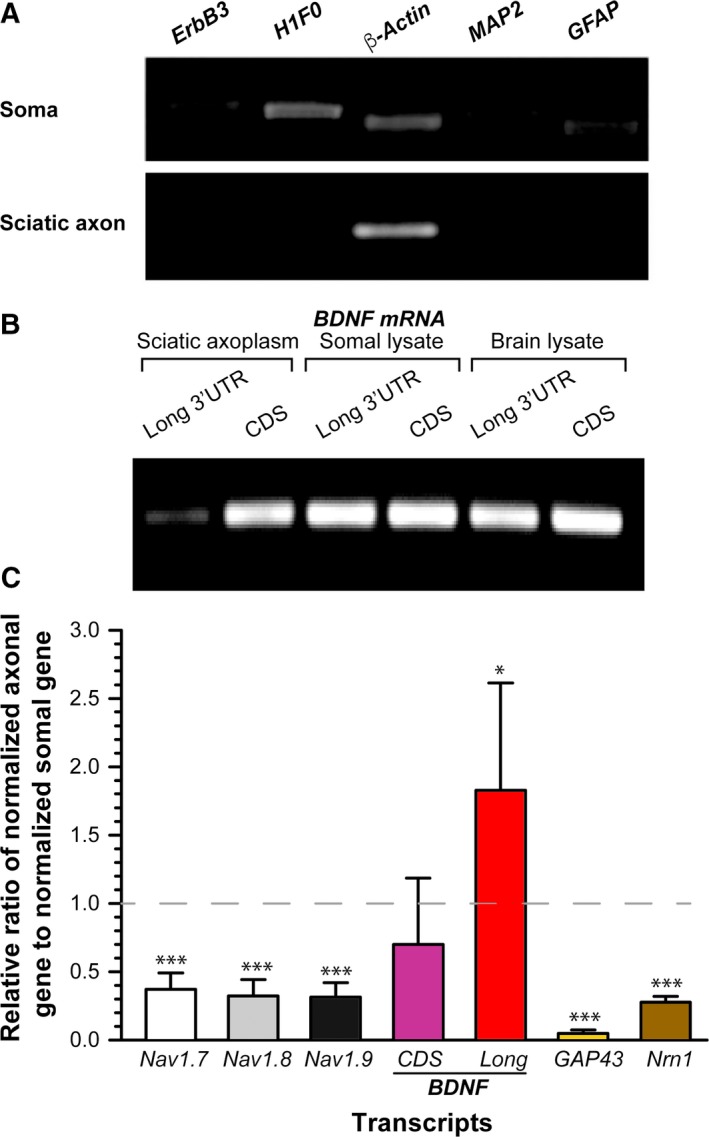
Endogenous *BDNF* mRNA with a long 3′ UTR is differentially distributed in distal axons of sensory neurons. (A) Extended RT‐PCR shows that neither cell body (soma) restricted (*MAP2*,* H1F0*), nor glial (*GFAP*,* ErbB3*) mRNAs were amplified from sciatic axon cDNAs. (B) RT‐PCR products of RNAs extracted from indicated tissues show that a long 3′ UTR variant of *BDNF* mRNA was weakly expressed in the sciatic axon. (C) Relative ratio (R.R) of indicated mRNA levels in axon to the corresponding mRNAs in soma. The indicated genes in axon and soma were first normalized to axonal and somal *amphoterin* mRNAs, respectively, and data are presented as the axonal gene/somal gene ratio: R.R = (gene^axon^/amphoterin^axon^)/(gene^soma^/amphoterin^soma^). The SD was calculated from the average of at least three different biological samples (*P < 0.05, ***P < 0.001 compared to somal level by Student's *t*‐test).

Five hundred nanograms of RNA was reverse‐transcribed to cDNA using an iScript™ cDNA Synthesis Kit (Bio‐Rad) in accordance with the manufacturer's instructions. To confirm the purity of the extracted axoplasmic RNA, an extended RT‐PCR method (40 cycles) was utilized to check contamination from cell bodies and non‐neuronal cells, including glial cells. RNA was considered as highly enriched axoplasmic RNA and used for further studies only when axoplasmic RNA samples were not detected for cell body‐restricted *microtubule‐associated protein 2* (*MAP2*) and *H1 histone family member 0* (*H1F0*), non‐neuronal *glial fibrillary acid protein* (*GFAP*) and *receptor tyrosine‐protein kinase ErbB family‐3* (*ErbB3*) mRNAs (Fig. 1A). In addition, the presence of *β‐actin* mRNA in the sample was indicative of the presence of mRNA in the extraction.

### Droplet digital PCR (ddPCR)

ddPCR is a technological refinement of the conventional PCR method that uses a water–oil emulsion droplet system to partition a PCR sample into nanoliter‐size samples and encapsulate them into oil droplets. The protocols that were followed were as described previously [18]. Briefly, cDNAs of DRG (soma) or sciatic axoplasm were mixed with the QX200 ddPCR EvaGreen Supermix (Bio‐Rad) and specific primers for rat *Nav1.6*,* Nav1.7*,* Nav1.8*,* BDNF long 3′UTR*,* BDNF coding region*,* neuritin 1* (*Nrn1*), *Gap43* and *amphoterin* (also called *Hmgb1*) mRNAs (Integrated DNA Technologies, Coralville, IA, USA). The PCR reactions were then placed in a droplet generator system that partitions the reaction into 20 000 nL sized oil‐based droplets followed by PCR amplification in accordance with the manufacturer's instructions. Copy numbers of target mRNAs in units of copies per μL input were determined using quantasoft (Bio‐Rad) to provide an absolute quantification of target DNA copies per input sample. To account for copy number variations in axoplasmic and somal RNA samples between biological samples, target mRNA copy numbers were normalized with the copy numbers of *amphoterin* mRNA because this was previously shown to be constitutively transported into axons of adult DRG neurons without changes in expressed mRNA level upon nerve injury 18, 19. The relative ratio (R.R) of target mRNA levels in axon to those in soma were calculated as: R.R = (gene^axon^/amphoterin^axon^)/(gene^soma^/amphoterin^soma^).

### DNA constructs

**Figure 2:**
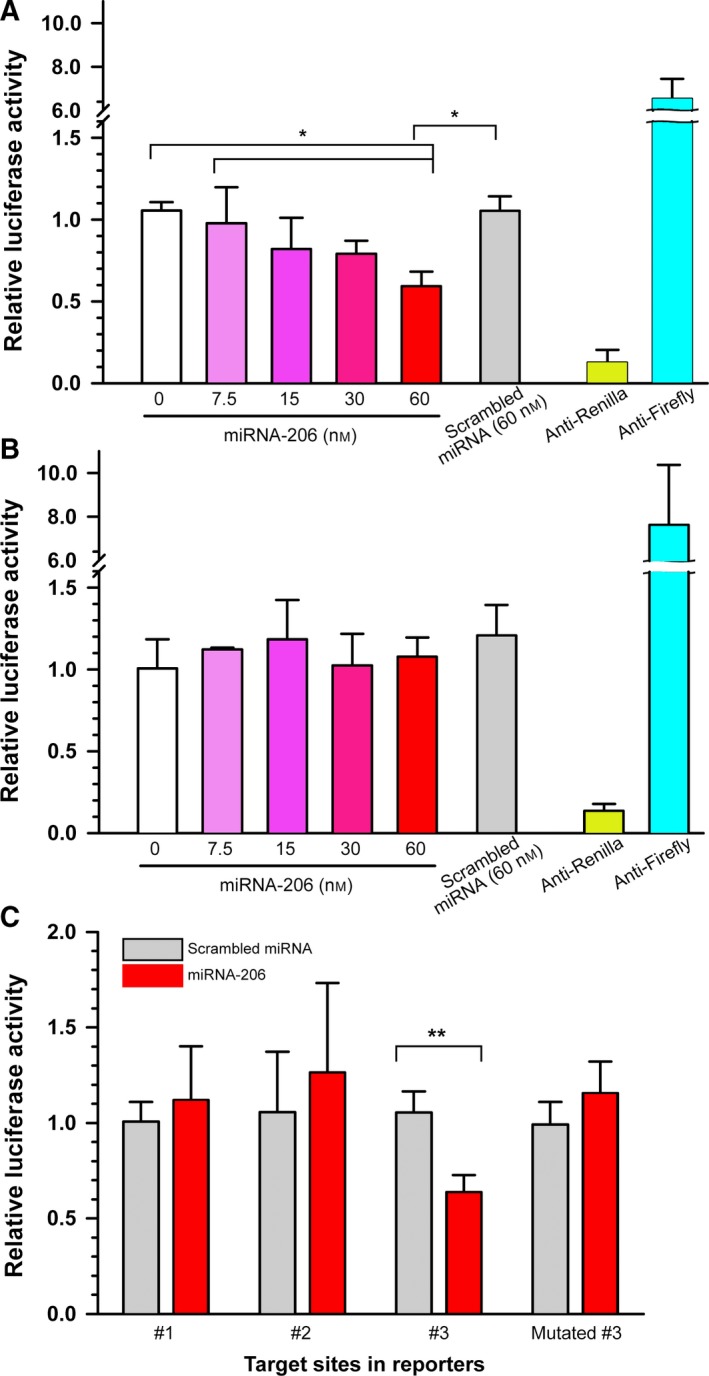
miRNA‐206 specifically downregulates *BDNF* mRNA with a long 3′ UTR. (A) Dual‐luciferase assays showed a significant reduction in the luciferase activities of vectors fused to a long 3′ UTR by miRNA‐206. *P < 0.05 by one‐way ANOVA with Bonferroni post‐hoc comparisons. Error bars represent the SD (*n* = 3). (B) miRNA‐206 failed to affect the luciferase activity of vectors fused to a short 3′ UTR. One‐way ANOVA with Bonferroni post‐hoc comparisons. Error bars represent the SD (*n* = 3). (C) Transfection of miRNA‐206 reduced the luciferase activities of vectors containing #3 target site but failed when the seed sequences of the #3 sites were mutated, revealing the target specificity. **P < 0.01 by Student's *t*‐test, Error bars represent the SD (*n* = 4).

3′ UTR variants (a long and a short 3′ UTR) of *BDNF* mRNA were PCR amplified and cloned into Renilla luciferase reporter (Promega, Madison, WI, USA) between the *Not*I and *Xho*I restriction sites. To generate Renilla luciferase reporter containing predicted target sites of miRNA‐206 (Target site #1, #2, and #3), as well as the mutated target site #3 (Fig. 2), the sense and antisense sequences of each of the target sites were engineered to contain terminal sticky ends of the *Not*I and *Xho*I restriction sites and dimerized to be directly ligated to backbone of Renilla constructs pre‐digested with restriction enzymes.

### Dual‐luciferase reporter assay

Luciferase assays were performed in F‐11 cells (derived from the fusion between mouse neuroblastoma cell line N18TG2 with embryonic rat DRG neurons) using the Dual‐Luciferase Reporter 1000 Assay System (Promega). Briefly, F‐11 cells were plated at a density of 8000 cells/cm^2^ on 6 cm culture dishes and co‐transfected with internal control (firefly luciferase reporter) and experimental Renilla luciferase reporters using Lipofectamine 2000 (Invitrogen). Twenty‐four hours after transfection, the F‐11 cells were replated at a density of 5000 cells/cm^2^ in a 24‐well plate and incubated for 24 h before being transfected again with either scrambled miRNA, firefly or Renilla DsiRNAs (Integrated DNA Technologies), miR‐206 mimic (Invitrogen). DsiRNA is an siRNA control for the effective knockdown of luciferase genes and miRNA mimic is chemically modified miRNA‐like RNA that is designed to copy the functionality of mature endogenous miRNA upon transfection. Both firefly and Renilla luciferase activities were measured sequentially from the cell lysate and the normalized values (Renilla activity/firefly activity) were used for analysis to minimize experimental variability between replicates.

### DRG primary neuronal culture in the modified Boyden chamber

The modified Boyden chamber was used to physically separate axonal and somal compartments 19, 20, 21. Briefly, 8 μm tissue culture inserts were placed in six multiwell culture plates and doubly coated with poly‐l‐lysine (Sigma, St Louis, MO, USA) and laminin (Millipore, Billerica, MA, USA). DRG neurons were dissociated in collagenase and washed multiple times with DRG culture media containing 5% horse serum, 5% fetal bovine serum, 1 × Hepes, 2 mm l‐glutamine, 1 × N1 supplement and 10 μm AraC in Dulbecco's modified Eagle's medium/F‐12 (Invitrogen). After transfection with miRNA‐206 using the 4D‐Nucleofector system (Lonza, Allendale, NJ, USA), cells were plated onto the tissue inserts and incubated for 3 days in culture at 37 °C with 5% CO_2_.

### Immunoblotting

Axonal total proteins were isolated from the tissue culture inserts by scraping the bottom surface of the membrane with a cell scraper and dispersed in modified radioimmunoprecipitation assay buffer supplemented with phenylmethanesulfonyl fluoride, a protease cocktail and a phosphatase inhibitor (Sigma). Somal proteins were also processed by scraping the upper membrane. Lysates were mixed with 5 × loading buffer (denaturing buffer), boiled for 5 min and then immediately placed on ice to denature the proteins and then loaded onto 12% SDS/PAGE gels followed by transferring onto a poly(vinylidene difluoride) membrane. After blocking in 5% non‐fat milk in Tris‐buffered saline with 0.1% Tween‐20, the membrane was probed first with anti‐BDNF (dilution 1 : 1000; Bioss Inc., Woburn, MA) overnight at 4 °C on a shaker. After washing extensively in Tris‐buffered saline with 0.1% Tween‐20, the membrane was incubated in a secondary antibody conjugated to horseradish peroxidase (dilution 1 : 3000; Cell Signaling Technology, Beverly, MA, USA). Horseradish peroxidase activity was visualized using an enhanced chemiluminescence prime western blotting detection reagent (GE Healthcare, Little Chalfont, UK). After developing the membrane to X‐ray film, the membrane was extensively washed and probed with anti‐GAPDH (dilution 1 : 3000; Cell Signaling). GAPDH served as an internal control for normalization.

### Northern blotting

Five micrograms of total RNA was analyzed for each sample on 1% agarose gels followed by transfer to a nylon membrane. As a control, 5 μg of somal RNA was incubated with 100 μg of RNase A (Qiagen, Valencia, CA, USA) at 37 °C for 30 min prior to electrophoresis. Northern blotting was performed as described previously 22. Briefly, after UV crosslinking, the blot was hybridized with biotin‐labeled antisense probe specific for BDNF coding region and detected by the Chemiluminescent Nucleic Acid Detection Module (Pierce, Rockford, IL, USA). Biotin‐labeled probe was synthesized using a Biotin RNA Labeling Mix (Roche, Basel, Switzerland) during *in vitro* transcription with the Riboprobe® *In Vitro* Transcription Systems (Promega).

### Fluorescence recovery after photobleaching (FRAP)

Myristoylation tag (*myr*) was added to the GFP or mCherry to limit cellular free diffusion because *myr* tag will target the newly synthesized reporter protein to the adjacent membrane. DRG neurons were transfected with either ^*myr*^eGFP‐BDNF long 3′ UTR, ^*myr*^mCherry‐BDNF short 3′ UTR construct, or both, using the 4D‐Nucleofector system (Lonza) and plated on glass‐bottom 35 mm culture dishes that were doubly coated with poly‐l‐lysine and laminin. Transfected DRG cultures were analyzed 48–72 h after transfection for intra‐axonal eGFP or mCherry fluorescence. A LSM 880 confocal microscope fitted with an environment chamber (Carl Zeiss, Oberkochen, Germany) was used for photobleaching and monitoring recovery of fluorescence. Axons longer than 400 μm in length from the cell body were carefully selected for the FRAP experiments to minimize the diffusion of GFP or mCherry from the cell body during recovery. Terminal axons were imaged every 30 s for 10 min with a 488 nm or a 594 nm laser to establish a baseline fluorescence intensity of eGFP or mCherry, respectively (prebleach). A region of interest (ROI) of terminal axons was exposed to 100% power of the 488 nm or the 594 nm laser for 35 repeats for photobleaching (bleaching). The recovery of fluorescence within the photobleached ROI was then monitored by acquiring images every 60 s under the same 488 nm or 594 nm laser sets as in the prebleach for a total of 30 min (postbleach). Cycloheximide (100 μg·mL^−1^) was added as a translational inhibitor to ensure that fluorescence recovery depends on local protein synthesis. Recovery was quantified from *n* = 7 neurons using imagej (NIH, Bethesda, MD, USA) to calculate average pixels per μm^2^ in the ROIs of the raw confocal images.

### Statistical analysis

prism, version 5 (GraphPad Software Inc., San Diego, CA, USA) was used for all of the statistical analyses. ddPCR and the luciferase assay were analyzed using Student's *t*‐test and one‐way ANOVA with Bonferroni post‐hoc comparisons, respectively.

## Results

### *BDNF* mRNA with a long 3′ UTR is present at low abundance in distal axons of adult rat sensory neurons

Two different 3′ UTR variants of *BDNF* mRNA have been identified and the 3′ UTR has been reported to be responsible for their subcellular localization pattern 1, 10, 11, 12. However, most of the earlier studies were performed in dendrites of central nervous system neurons, propmpting debate about the abundance of localized *BDNF* mRNA with a long 3′ UTR in distal axons. Therefore, we examined the abundance and localization of endogenous *BDNF* mRNA with a long 3′ UTR in the distal axons of adult rat sensory neurons. First, to re‐evaluate subcellular localization of the long 3′ UTR variant of *BDNF* mRNA in distal axons of DRG neurons *in vivo*, sciatic nerve axoplasm was mechanically extracted from the nerve of adult rats using the mechanical squeezing method that we optimized previously 17. To determine the purity of the axoplasmic RNA, extended (40 cycles) RT‐PCR was carried out to assess glial and cell body contamination (Fig. 1A). *H1F0* and *MAP2* mRNAs are shown to be excluded from axons 20 and thus are used to determine the contamination of cell body‐restricted mRNAs into axons. Levels of glial‐specific mRNAs (*GFAP* and *ErbB3*) were also tested to ensure minimal contamination from Schwann or satellite cells. *β‐actin* was included to confirm the presence of mRNA in the extracted samples (Fig. 1A) because its presence in both the axon and soma of sensory neurons has been demonstrated previously 18, 19, 20, 21. As shown in Fig. 1A (bottom), extended RT‐PCR using an equivalent amount of cDNAs from sciatic nerve (axon) and DRG (soma) only demonstrated a positive PCR product for *β‐actin*, whereas glial cell or cell body‐restricted mRNAs were undetectable, indicating highly enriched axonal RNAs.

After determining the purity, the presence of *BDNF* mRNA with a long 3′ UTR within the distal axons was confirmed by semiquantitative RT‐PCR using specific primers for the long 3′ UTR (Fig. 1B). Consistent with previous studies showing the presence of a long 3′ UTR variant of *BDNF* mRNA in the dendrites of central nervous system neurons 12, we detected *BDNF* mRNA with a long 3′ UTR in all samples, including sciatic nerve axoplasm. The lower intensity of the PCR amplicon for *BDNF* mRNA with a long 3′ UTR from sciatic axoplasm also suggested a relatively low abundance of the transcript in axons.

To determine whether axonal *BDNF* mRNA with a long 3′ UTR is differentially distributed in axons and cell bodies, we used droplet digital PCR technology. Similar to previous studies 10, 23, the relative number of copies for the long 3′ UTR to the total *BDNF* in cell body sample was calculated as a ratio of 1:4 (2.8 copies vs. 11.4 copies). Next, to gain an estimate of the relative abundance of *BDNF* mRNA with a long 3′ UTR in axons to that in cell bodies, the number of copies of *BDNF* mRNA was first normalized to that for *amphoterin* mRNA to account for varying amounts in axons and cell bodies between experimental samples 18, 19. We then contrasted the level of axonal *BDNF* mRNA to that of somal *BDNF* mRNA. We also included specific primer sets for peripheral sensory neuron‐specific sodium channels including Nav1.7, Nav1.8 and Nav1.9 isoforms as positive markers for sensory neurons 24, 25. As shown in Fig. 1C, the relative level of axonal *BDNF* transcript with a long 3′ UTR was approximately 1.8‐fold higher compared to that measured in cell body, suggesting a preferential localization of *BDNF* mRNA with a long 3′ UTR to distal axons. Note that the lower ratio could be interpreted as either the absolute copy number or relative abundance of target mRNA being lower compared to the soma. Both *Gap43* and *Neuritin* (*Nrn1*) mRNAs have been shown to localize to the axons of sensory neurons 20, 21, 26, 27, 28 and so the relatively low ratio indicates that the soma level is relatively larger than those of the axons. Taken together, these results suggest that the long 3′ UTR enables preferential localization of *BDNF* mRNA into axons.

Although our cRNA probe hybridization protocol to visualize the endogenous *BDNF* mRNA with a long 3′ UTR plagued the fluorescence *in situ* hybridization signal in our hands, we carried out northern blot analyses of total RNAs from DRG soma and sciatic axoplasm using RNA probes derived from the *BDNF* coding region. Both the long and short *BDNF* mRNAs were present in all examined samples (Fig. S1). In this experiment, we also included RNase A‐treated somal RNA sample and used as a negative control to assess the signal specificity.

### The long 3′ UTR variant of *BDNF* mRNA is differentially regulated by miRNA‐206

Although two 3′ UTR variants of *BDNF* mRNA are reported to confer distinct subcellular distribution of the isoforms and functions in neurons 12, we are only beginning to understand the scope of molecular mechanisms that enable subcellular region‐specific post‐transcriptional regulation of mRNA isoforms, which contributes to the functional diversity between subcellular locales within a neuron. Targeting of brain *BDNF* mRNAs with miRNA‐206 was first demonstrated in Tg2576 Alzheimer disease transgenic mice 16. Because a longer 3′ UTR contains more chances of having a miRNA target site(s), we hypothesized that *BDNF* mRNA with a long 3′ UTR variant present in distal axons of sensory neurons is specifically subjected to a differential regulation by miRNA‐206, compared to a short variant.

**Figure 3:**
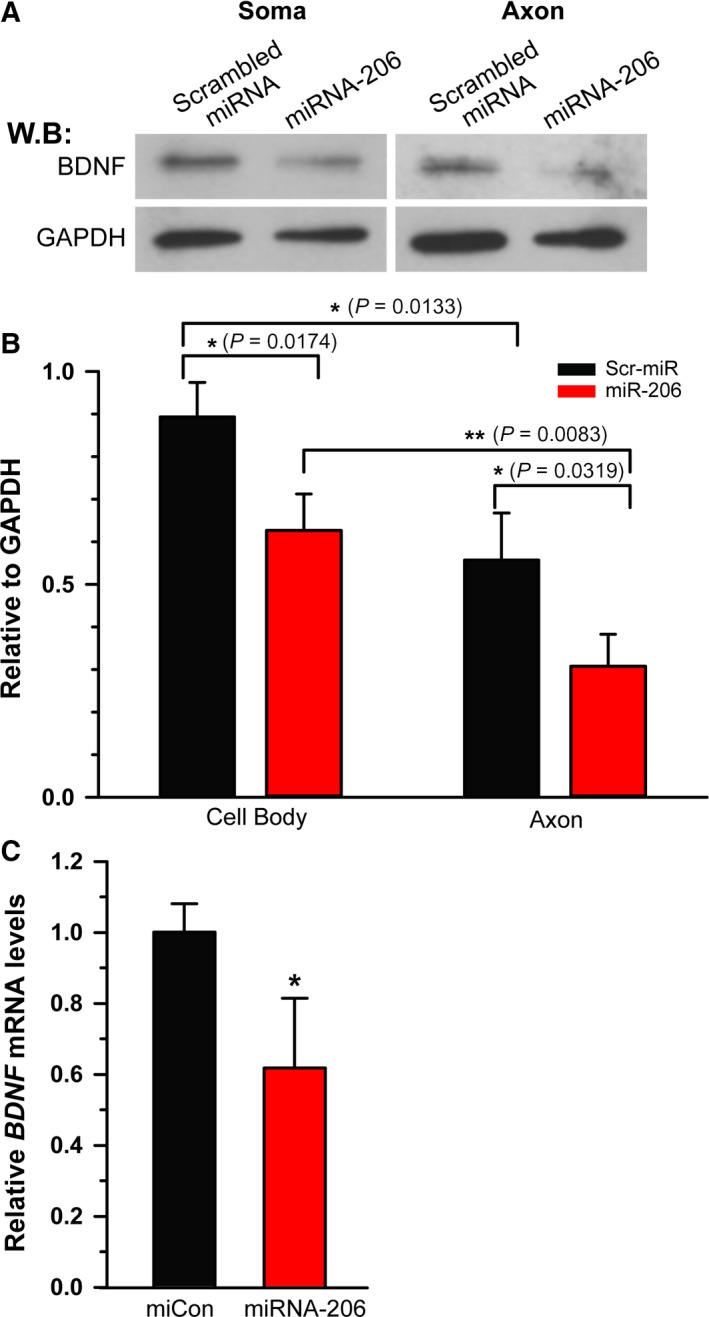
miRNA‐206 decreases BDNF protein in sensory neurons. (A) Western blot analysis was performed on somal and axonal lysates obtained from transfected DRG neurons cultured for 3 days and probed with antibodies to BDNF and GAPDH. GAPDH was included as a loading control. (B) Densitometric analysis showed a significant decrease in both somal and axonal BDNF expression. Note that the decreased level of BDNF expression by miRNA‐206 in axon was statistically lower compared to that in soma. *P < 0.05, **P < 0.01 by Student's *t*‐test. Error bars represent the SD (*n* = 3). (C) Quantification of *BDNF* mRNA levels in miRNA‐206 transfected DRG neurons was measured by qRT‐PCR and normalized to *GAPDH* mRNA. Consistent with the reduction in BDNF protein, the significant reduction in *BDNF* mRNA level indicted miRNA‐206‐mediated regulation of BDNF expression at the mRNA level. *P < 0.05 by Student's *t*‐test. Error bars represent the SD (*n* = 3).

To directly contrast potential miRNA‐206 target sites predicted in a long 3′ UTR variant vs. a short 3′ UTR, two Internet‐based algorithms, *TargetScan* and *miRanda*, were used and found three vs. one putative target sites of miRNA‐206 in the long and short 3′ UTR, respectively (Fig. S2). To test the target specificity of miRNA‐206, we used luciferase reporters fused to either a short or long 3′ UTR variants of *BDNF* mRNA. Transient overexpression of cells with miRNA‐206 significantly reduced the luciferase activities in the cells transfected with the vectors containing a long 3′ UTR variant at a concentration of 60 nm (Fig. 2A). By contrast, when the cells were transfected with the reporter vector bearing a short 3′ UTR, overexpression of miRNA‐206 failed to change the luciferase activity (Fig. 2B). To test further the specificity of the target sites within a long 3′ UTR, we repeated the assay with luciferase vectors bearing the individual putative target sites. As shown in Fig. 2C, only the target site #3 showed a significant reduction in the luciferase activity by miRNA‐206. However, when the seed sequence of the target site #3 was mutated, miRNA‐206 did not change luciferase activity. These results indicated that miRNA‐206 differentially regulates a specific *BDNF* mRNA variant via recognition of the target site #3 present only in the long 3′ UTR. It is worth noting that we detected a significant reduction in *BDNF* mRNA in response to miRNA‐206 overexpression, which is consistent with the reduction in BDNF protein (Fig. 3B), suggesting that miRNA‐206 regulates BDNF expression, at least in part, via an effect on mRNA stability in DRG neurons. Taken together, local expression of *BDNF* mRNA variant with a long 3′ UTR can be specifically downregulated by miRNA‐206 in distal axons without affecting the counterpart with a short 3′ UTR in the cell body.

### miRNA‐206 decreases BDNF protein expression in cultured adult sensory neurons

To investigate whether miRNA‐206 enables a differential spatial regulation of BDNF expression at a subcellular level in a neuron, we used the modified Boyden insert to physically separate the axonal compartment and cell body compartment. DRG neurons transfected with miRNA‐206 were cultured for 3 days on the tissue culture inserts doubly coated with poly‐l‐lysine and laminin. The axonal lysate and cell body lysates were obtained by scraping the bottom and top of the insert membrane, respectively. After the purity analysis, the somal and axonal lysates were subjected to western blot analysis to monitor BDNF protein levels (Fig. 3A). Transfection efficiencies were often very low to moderate (an average transfection efficiency of < 40–50%) in post‐mitotic cells such as mature DRG neurons derived from adult rats. Therefore, measurement of BDNF expression level by western blot analyses on total lysate may underestimate the true regulatory activity of the interfering miRNA‐206 that is spatial‐specific. Fortunately, the transfection of miRNA‐206 into DRG neurons significantly decreased BDNF protein levels by 30.1 ± 3.4% and 44.9 ± 6.1% in the cell bodies and axons, respectively, compared to those transfected with control scrambled miRNA (Fig. 3B). This difference was found to be significant (*P *=* *0.0218) on statistical analysis by an unpaired two‐tailed Student's *t*‐test. Taking in account the moderate transfection efficiencies of DRG neurons with miRNA‐206, this result implies a plausible underestimation of differential regulation of axonal BDNF expression, and a more robust effect of miRNA‐206 might be linked to its differential regulation of BDNF expression in the axonal compartment compared to that in cell body compartment. Consistent with the reduction in BDNF protein, we detected a significant reduction in total *BDNF* mRNA levels in response to miRNA‐206 transfection (Fig. 3C), suggesting that miRNA‐206 downregulates BDNF expression, at least in part, via mRNA destabilization. Taken together, these data show that miRNA‐206 differentially targets *BDNF* mRNA variant with a long 3′ UTR from the counterpart with a short 3′ UTR.

## Discussion

In the present study, we found that a long 3′ UTR variant of rat *BDNF* mRNA, which is present in distal axons of sensory neurons, is differentially regulated by miRNA‐206 compared to its shorter 3′ UTR counterpart in subcellular regions of sensory neurons. The results suggest that transcripts with a longer 3′ UTR potentially harboring more miRNA‐binding sites can provide a precise molecular mechanism for spatially differential downregulation in a subcellular region‐specific manner.

*BDNF* mRNAs are polyadenylated at two alternative poly(A) sites, leading to two distinct 3′ UTR variants, a short form (0.35 kb) and a long 3′ UTR (2.9 kb), which are reported to be responsible for the distinct subcellular localization 1, 10, 11, 12. An *et al*. 12 previously showed that *BDNF* mRNA containing a short 3′ UTR variant is restricted to the soma and suggested that the localization *cis*‐element(s) reside within the distal segment of the long 3′ UTR of *BDNF* mRNA. However, the substantive *cis*‐elements are still a matter of debate because the results of other studies suggest that dendritic targeting elements are found in the short 3′ UTR 29, 30. Consistent with this notion, we detected both the long and short *BDNF* mRNAs in all of the samples examined, including sciatic nerve, by northern blot analysis of total RNA (Fig. 1 and S1). Furthermore, our data from the FRAP analyses revealed a significant recovery after photobleaching in DRG neurons transfected with fluorescent reporter fused with a short 3′ UTR of *BDNF* mRNA, as well as that fused with a long 3′ UTR (Fig. S3), although the recovery level was relatively lower with the short 3′ UTR compared to that with the long 3′ UTR. Treating cells with translation inhibitors such as cycloheximide prior to bleaching blocked the fluorescence recovery, indicating that the recovery of fluorescence signals after photobleaching is a result of the local protein synthesis of localized reporter mRNAs. This could be explained by many factors, most significantly the differential degree of targeting capability of multiple *cis*‐elements that control the mRNA localization in axons. Indeed, several previous studies suggest the presence of multiple *cis*‐elements within the 3′ UTR of transcripts that have a differential degree of axonal targeting capability 10, 30, 31. Additionally, different experimental conditions, including differences between neurons derived from the central nervous system and the peripheral nervous system, may cause such a discrepancy, with the differential expression level of *trans*‐acting factors such as RNA‐binding proteins contributing to the complexity of the regulation of mRNA transport and translation at post‐transcriptional levels. Future studies will be essential to confirm the degree of localization capability between multiple motifs in the 3′ UTR.

miRNAs are short non‐coding RNAs that regulate translation of mRNAs by binding to their target sequences usually within the 3′ UTR in a sequence‐dependent manner. Consistent with previous studies 16, 32, our luciferase reporter assays and western blot analyses showed that the expression of *BDNF* mRNA in distal axons of sensory neurons is specifically downregulated by miR‐206. miRNA‐206 is one of the most studied miRNAs and was originally considered to be vertebrate muscle specific 33, 34. However, relatively recent studies have demonstrated its expression in non‐muscle tissues, including fat, liver and breast, as well as in the nervous system with respect to Alzheimer's disease, amyotrophic lateral sclerosis, cerebral ischemia, schizophrenia and regenerating nerves 16, 35, 36, 37, 38, 39, 40. This suggests potential functions of miRNA‐206 in the nervous system in addition to the developmental and pathological roles in muscle cells 34. Given that both the short and long 3′ UTR variants of *BDNF* mRNA are present in distal axons, miRNA‐206 may be able to distinguish the long 3′ UTR of *BDNF* mRNA from the short form in the process of translational control in distal axons. It is not clear yet why these two 3′ UTR variants of *BDNF* mRNA are localized to distal axons and regulated differentially by miR‐206 because our preliminary data failed to reveal biological effects of miRNA‐206 transfection on DRG neurons derived from adult rats (data not shown). However, it is plausible that the long 3′ UTR form of *BDNF* transcript encoded a protein with novel functions. The newly synthesized protein from the long variant of *BDNF* mRNA within axons could act as a retrograde injury signal, as shown in previous studies 41, 42, 43, 44, 45, 46. A major challenge to understanding whether locally synthesized BDNF protein in axons has a novel biological role in neurons involves monitoring the trafficking of locally synthesized protein in axons. Also, it will be interesting to determine what happens to BDNF local synthesis when the negative regulation by miR‐206 is blocked.

It is estimated that more than 50% of mammalian transcripts contain alternative poly(A) sites that can lead to different 3′ UTR variants 47. Several previous studies showed that different variants of the same transcript are differentially transported to axons, suggesting the presence of sequences or elements conferring axonal targeting within the 3′ UTR. However, it is unclear how the translation of these variants is differentially regulated from their counterparts with a shorter 3′ UTR. The work reported in the present study suggests that variant‐specific sequences contain miRNA‐binding sites or sites for RNA‐binding proteins, providing a unique ability for precise control of the temporal and spatial regulation of BDNF protein upon neuronal activation.

## Author contributions

SS, HHK and SY designed the experiments. SS and SY wrote the manuscript. SS, HHK and PP extracted the RNA. SS and MP performed the sequencing and bioinformatics analyses. SS, MP and HHK performed the reporter generation, luciferase activity assays and FRAP experiments. SL performed the ddPCR.

## Conflicts of interest

The authors declare no conflict of interest.

## Supporting information


**Fig. S1.** *BDNF* mRNAs with a short and a long 3′ UTR were present in sciatic nerve.
**Fig. S2.** Schematic of 3′ UTR variants of *BDNF* mRNAs, a long 3′ UTR and a short 3′ UTR.
**Fig. S3.** BDNF 3′ UTR is sufficient for localization and translation of a reporter mRNA in axons of sensory neurons.
