# Supplementary material for: Differential regulation of brain‐derived neurotrophic factor (BDNF) expression in sensory neuron axons by miRNA‐206
Source: FEBS Open Bio. 2019 Jan 16;9(2):374–83. doi: 10.1002/2211-5463.12581 (PMC6356166; doi:10.1002/2211-5463.12581)
Supplement: Supplementary file 1 — Fig. S1. BDNF mRNAs with a short and a long 3′ UTR were present in sciatic nerve. Fig. S2. Schematic of 3′ UTR variants of BDNF mRNAs, a long 3′ UTR and a short 3′ UTR. Fig. S3. BDNF 3′ UTR is sufficient for localization and translation of a reporter mRNA in axons of sensory neurons. [file FEB4-9-374-s001.pdf]

Supplementary Figure S1.

***BDNF* mRNAs with a short and a long 3'UTRs were present in sciatic nerve.**

Both a long and a short 3'UTR variants of *BDNF* mRNA were detected in sciatic axon and soma by northern blot using coding region specific probes. Somal RNA pretreated with RNase A was also included as a control. *Arrows* and *arrowheads* indicate *BDNF* mRNA with a long 3'UTR and a short 3'UTR, respectively.

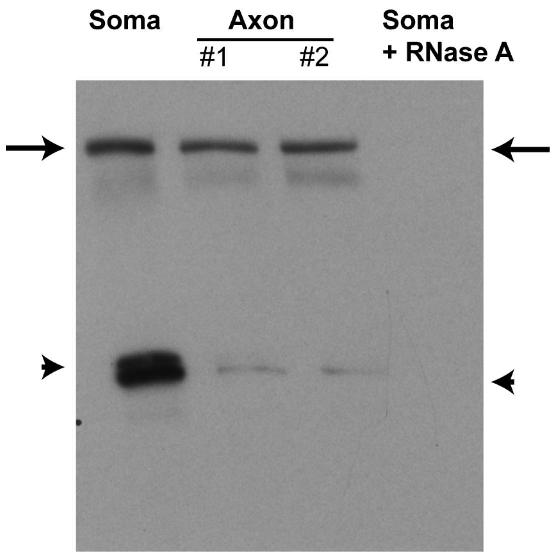

Supplementary Figure S2.

Schematic of 3'UTR variants of *BDNF* mRNAs, a long 3'UTR and a short 3'UTR (*Top*) and 3 predicted target positions of the rat *BDNF* 3'UTR are aligned with rat miRNA-206 (*Bottom*). Asterisks indicate predicted miRNA-206 target sites.

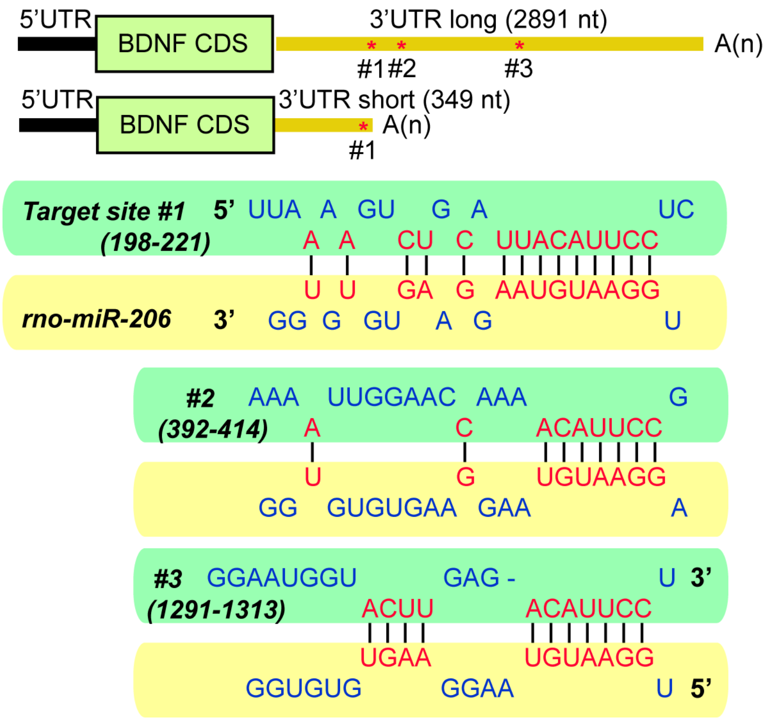

Supplementary Figure S3.

BDNF 3'UTR is sufficient for localization and translation of a reporter mRNA in axons of sensory neurons.

Quantifications for multiple time-lapse sequences from FRAP experiments with DRG neurons transfected with ^{myr}GFP-BDNF long 3'UTR (Top) or ^{myr}mCherry-BDNF short 3'UTR (Bottom) are shown as normalized average signal intensity relative to pre-bleach. Data are represented as average ± SEM (n=7 neurons over >3 separate experiments for each construct; **p<0.01, ***p<0.001 for indicated time points versus t=0 min by repeated measures ANOVA with *Bonferroni post hoc* comparisons.

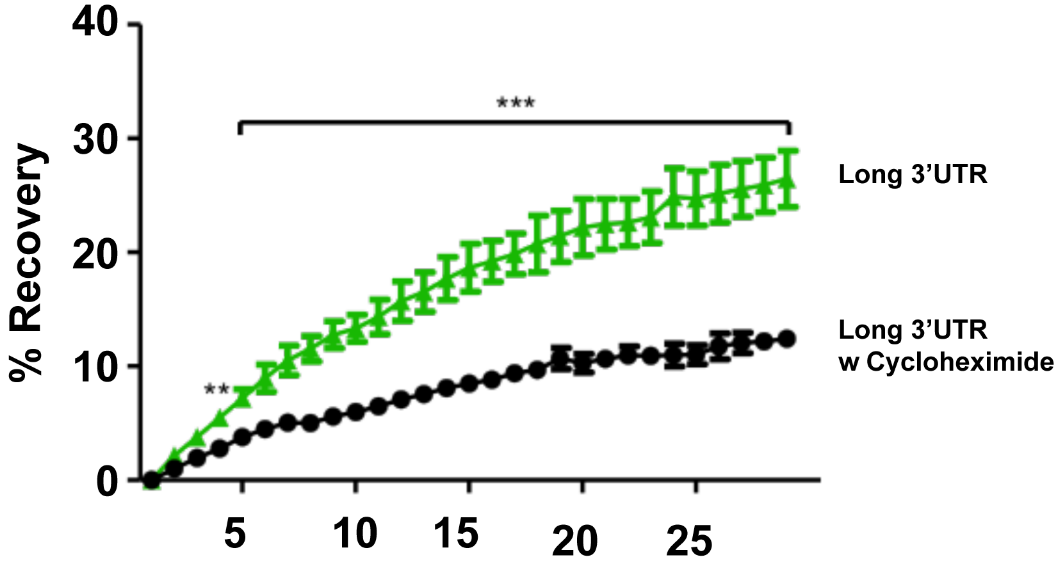
40
30
20
10
0
% Recovery
***
**
5
10
15
20
25
Long 3'UTR
Long 3'UTR
w Cycloheximide

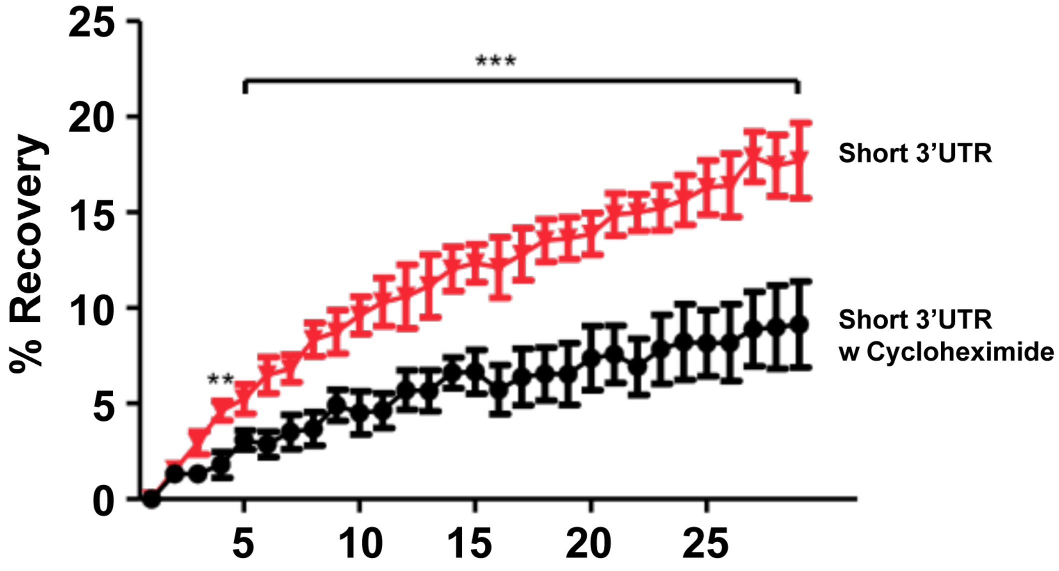
25
20
15
10
5
0
% Recovery
***
**
5
10
15
20
25
Short 3'UTR
Short 3'UTR
w Cycloheximide
